# Selling the Return on Investment for Digital Health

**DOI:** 10.1089/tmr.2024.0069

**Published:** 2025-01-31

**Authors:** Judd E. Hollander, Gregg Meyer, Ralph Derrickson, Baligh Yehia, Anne Docimo

**Affiliations:** ^1^ SVP Healthcare Delivery Innovation, Jefferson Health, Philadelphia, Pennsylvania, USA.; ^2^ President of Community Division and Executive Vice President Value-Based Care Mass General Brigham, Boston, Massachusetts, USA.; ^3^ Creative Destruction Lab Computational Health Stream; Washington, USA.; ^4^ Jefferson Health, Wayne, Pennsylvania, USA.; ^5^ Board of Directors, Chief Medical Officer United Healthcare, NCQA, Minneapolis, Minnesota, USA.

**Keywords:** finance, digital health, investment

## Abstract

**Background:**

Advancing digital health requires a realistic conversation that moves past innovation and evaluates digital tools the same as any other device being introduced into the health system. There needs to be a focus on return on investment.

**Methods:**

As part of a symposium, we presented hypothetical pitches to an expert panel. The experts include representatives from health systems, payers, and investors. The pitches were related to remote patient monitoring, tele-triage in the emergency department, and comprehensive in-patient telemedicine program including virtual sitting and e-nursing.

**Results:**

Although each pitch led to a different discussion, there was uniform agreement that health systems should focus on whether the proposal helps solve an institutional problem; the payment model in which the product can be used (value-based, fee-for-service, or both) needs to be identified; fitting the new product into preexisting workflow (included electronic health system integration) is critical; there needs to be an understanding of whether patients and providers engage with it; and there needs to be a clear return on investment.

**Discussion:**

Navigating complex decision-making in health care requires a blend of strategic foresight, practical considerations, and a deep understanding of organizational dynamics. Rather than a specific strategic plan focused on digital or virtual care, there should be a focus on the enterprise strategic plan and how can digital enable that.

## Introduction

Advancing digital health requires a realistic conversation that moves past innovation and “coolness” and begins to evaluate digital tools in the same manner as any other device being introduced into the health system.^1–3^ That focus is the return on investment (ROI).

In this article, we summarize a robust discussion between leaders within health systems, payers, investors, and venture capitalists using hypothetical pitches for products that are being presented to health systems. None of the pitches are a replica of a single product, but they are designed to reflect the type of pitch health systems often receive.

## The Panelists Are

Greg Meyer, MD, MSc, is a primary care physician and general internist. He is president of the community division and executive vice president value-based care at Mass General Brigham. Part of his portfolio includes overseeing value-based care from the delivery system perspective and the insurance company.

Ralph Derrickson is the former CEO of Carena, Inc., a company providing access to medical care, first working with large self-insured employers such as Microsoft and Boeing in the Seattle area and then it morphed from an on-site delivery model into a virtual delivery model starting in 2008.

Baligh Yehia, MD, MPP, MSc, is an infectious disease physician who is president of Jefferson Health, which is in South Jersey, Philadelphia, and Lehigh Valley regions covering 60,000 employees and 30 hospitals.

Anne Docimo, MD, is the Chief Medical Officer for United Healthcare and was the founding Chief Medical Officer for University of Pittsburgh Medical Center Health Plan. She is responsible for coverage decisions, policies, and all clinical programs and contracts like value-based care.

## Pitch #1: Remote Patient Monitoring Pitch

This pitch (Table 1) begins with “this is who we are, we’re really good at what we do, and we’ve grown a whole bunch of companies and made very successful exits, selling our company in the past.” Next, health systems often hear entrepreneurs telling us about the clinical condition that is targeting.

**Table 1.** Hypothetic but Somewhat Typical Pitch from Remote Patient Monitoring Vendors to Health Systems

Who we are
Entrepreneurs with successful exits
The disease is bad (DM, HTN, CHF, etc.)
We can help you solve this problem
We improve patient engagement and experience, clinical services, and health outcomes
Your return on investment will be great
CMS pays $50; we will only charge you something less than $50
We can integrate with your electronic health record via FHIR
You just tell us who to include, we will manage everything
We will use your Tax ID (TIN) and you supply a medical director to oversee your portfolio

CMS, Centers for Medicare and Medicaid Services.

Then health systems often hear about how the company can help solve this problem in terms of patient engagement and experience, and what clinical services they can provide, and how they think it will be good in terms of health outcomes. There is seldom patient-oriented clinical outcomes data.

In our experience, approximately 80% of the pitches received for remote patient monitoring (RPM) devices present how much money we can bill Medicare for the service and how much money the company will charge the health system. The person making the pitch often considers this small gap to be “profit” and tries to convince the health system that this will result in a large amount of revenue for the health system. When health systems inquire about integration with our electronic health record everyone, they are often told that this can be done, whether or not the company has done this with another client.

The most complicated part of the equation is around billing, where vendors often begin by saying will take care of everything from device supply, shipping, reclaiming devices, and medical care. However, health systems are routinely asked to do the billing since they have better rates. Also, usually required is the health system to provide medical oversight of the program.

### Panelists

There is a concern that companies often identify a very narrow clinical area and focus on this area with software and specific providers. This model would require health systems to slowly integrate all of those different narrow applications into a combined and aggregated care delivery model. This is not realistic.

Every health care system or medical group is tackling many different problems in many different ways-workflow changes, composition of the workforce, or technology. Using hemoglobin A1c as an example, some solutions are technology-based, some are via remote monitoring, some are via access to clinics, and some are more community-based interventions. It is really important to have a clear vision of the goal you are trying to achieve. Health systems desire solutions that can fill gaps, accentuate strengths, and help them achieve their goal.

Major considerations of health systems focused on (1) whether the proposal is an unsolicited pitch or was solicited specifically to help solve an institutional problem, (2) the payment model in which the product can be used (value-based, fee-for-service, or both), (3) how the products fit into preexisting workflow (included electronic health system integration), (4) whether patients and providers engage with it, and then (5) what is the actual ROI.

Sometimes a product sounds promising in isolation, but when compared with other potential expenditures, it might not be the highest priority. Panelists uniformly agree that for a health system or a payer, past successful exits are unimportant and may not even be considered a positive. Health systems want to partner with companies that will be around for a long period of time. Successful exits are really focused on wanting high-margin software revenue. They rarely account for the human capital or fixed costs associated with staffing models.

From an investor perspective, it is obviously important to look at the market and how it will grow? Will health systems be interested? Has the company generated outcomes data? Who are the key opinion leaders and is there a meaningful way to engage with the health systems?

With respect to the payers, the insurance company is going to be negotiating with someone (e.g., a health system) to provide service. Will use of this product allow the health system to have something that can be integrated in product design in a way that can differentiate the product? Many commercial insurers eventually cover anything that Centers for Medicare and Medicaid Services (CMS) covers. For RPM, if a physician decides to put a patient on RPM, payers pay the CMS rate as part of Medicare Advantage.

The bigger concern for groups of physicians that may want to coordinate RPM is that there needs to be a clinical care model that goes along with the RPM data. They need to set control limits for normal numbers and what the response should be when those numbers are out of those bounds. Payers would prefer that these devices be used in value-based contracts where health systems can be paid for performance or share in savings.

The most important thing to remember any time you’re looking to use devices in both pay-for-performance and value-based contracts (shared savings) is that the clinical and the financial models have to align. The piece of technology alone is not going to make as big difference as the clinical model.

## Pitch 2: Emergency Department Tele-Intake

This is the concept that you as a patient could go into the emergency department (ED) and instead of, or in addition to, being seen by the triage nurse, the patient is seen immediately by a clinician. That clinician will ask the patient a series of questions, do a video-based exam, write a note, and spend 1–2 min with the patient, analogous to many physician in triage models (Table 2). After the initial assessment, patients will either go to X-ray or phlebotomy. In this model, tests are being obtained while the patient is waiting to be seen by the provider.

**Table 2.** Hypothetic Pitch for an Emergency Department Tele-Intake Platform

ER’s are backed up due to boarding due to reduced hospital capacity
In many ER’s, more than 5% leave without being seen
Poor care
Lost revenue ($1000 per patient)
Physicians in triage model ensure patients can be seen shortly after arrival
Virtual triage/tele-triage/tele-intake
One clinician can see 15–20 patients per hour (at more than one hospital) and initiate orders
LWBS reduced on first day to under 1%
In a six-hospital system (300k ER visits)
Full installation and hardware costs are 117k
Licensing costs negotiable ($2/per census or 600k per year)
Staffing costs (16 h per day APP) at $80/h (including benefits) or $467k/year/APP or 1.4 m for 3 APPs for the 6 hospitals
Does not count reduced triage staff (can do without a triage nurse)
LWBS reduced 5% to 1% = 12k visits*1000 visit = 12 million

APP, advanced practice provider; LWBS, leave without being seen.

EDs are crowded. As a result, there is a cohort of patients who came to the ED to get care, but before they receive care, they hit their tolerance threshold and they leave without being seen (LWBS). Left without being seen rates commonly run around 3–5%. In some urban places that can run as high as 10–15%.

Patient who LWBS get poor or no care. From a business model perspective, this is lost revenue. Let’s assume that it’s $1000 per patient. The physician in triage model ensures the patients are seen right away. This potentially allows hospitals to capture a facility fee and maybe the professional fee as well.

In a virtual or remote model, a single remote clinician can see and evaluate 15–20 patients an hour. One remote clinician can be working at two or three hospitals, evaluating patients and initiating orders before transferring care to another clinician who will review the testing, reevaluate the patient, and make further management and disposition decisions. In every place that I know that it’s been tried, literally on day one the left without being seen rate is reduced to under 1%.^4^

In our hypothetical example of a six-hospital system with 300,000 ED visits, installation and hardware costs are about $120,000 per year. Licensing costs might be about $2 per person. That would be about $600,000 per year. Someone needs to staff the program. Let’s assume it’s an advanced practice provider (APP) for 16 h per day. At $80 an hour including benefits that would be about $467,000 per year. If three APPs would be needed for this model, it would be $1.4 million dollars in staffing costs.

If the left without being seen rate was reduced from 5% to 1%. That’s 12,000 visits at $1000 per visit. That is a nice ROI. What are your thoughts?

### Panelists

This model approaches care in a nuanced way rather than doing the soup-to-nuts approach. It finds discrete parts of the care pathway that could be enhanced by telehealth. In this particular example, there is almost no downside in a busy ED. Although this has a significant financial upside, this also improves quality and safety. Once the person’s engaged with the provider their care is underway. They’re less likely to LWBS and there have been some great saves, including getting a patient with an ectopic pregnancy to the operating room before they were able to get into an ED room and diagnosing cauda equina in a patient who received the magnetic resonance imaging (MRI) before they received a room.

From the payer side, could this be done before the patient comes to the ED? Could they be connected to a nurse line, where the patient can be told that they don’t need to even go to the ED. They can just be given an appointment.

Ideally, an overall program would want to integrate downstream so that we can actually plug patients into the ambulatory care setting at lower costs than sending them to the ED. This is better implemented in conjunction with a virtual primary care service to optimize ED diversion. Utilization within a population that either doesn’t have a primary care provider or has other psychosocial things that might drive their behavior for chronic conditions might be a successful overall strategy.

Telemedicine and other digital technology are really about workflows and operations. It’s a really important concept that even the best technology in the world isn’t going to make up for a bad workflow.

## Pitch #3: Inpatient Telemedicine

Imagine having a two-way camera in every room that you could use for whatever you want to use it for. You could use it for virtual sitting, virtual nursing, provider consultations, or virtually anything else (Table 3).

**Table 3.** Hypothetical Pitch for an Inpatient Telemedicine

Put two-way cameras in patient rooms
Sitters/safety observers
E-nursing
Provider consultations
Reduced transfers
Ancillary staff
Pharmacists
Case workers
Discharge planners

The most solid ROI from this approach is with safety observers or telesitting. Replacing 12 one-on-one bedside safety observers with a single remote safety observer has over a $1,000,000 ROI. We can then leverage that ROI, to include other uses, which do not have as strong of an ROI. In essence, telesitting is the breadwinner with halo effect because you can do other things without incremental cost.

Virtual nursing can help improve patient length of stay or provide a service to a location with a nursing shortage. It may allow better care experiences, better quality care, and be more efficiency although it alone does not always provide the clearest ROI.

Reduced travel for consultants between hospitals improves physician satisfaction. Physicians can just beam into rooms and do the consults. This is reimbursed for some programs, particularly for rural hospitals.

You can extend the reach of care managers or the discharge planners to perform a lot of complex discharge planning with family members facilitating safer more coordinated transitions to the home.

There are details that need to be worked out. We need to evaluate patient and provider acceptance of this approach. How does the staffing work? Do we include pharmacists, caseworkers, and discharge planners? What training do we need? What’s the leanest safe staffing model?

This looks promising and is a great investment if we leverage sitter savings to pay for equipment. There will be many workflows that may need to be redesigned.

## Considerations in the Health System Approach to Innovation

### What does partnership look like?

One of the common questions being asked is, How does a health system “partner” with a new company? Large medical centers are looking for true partnerships rather than just paying a vendor for new products. From the vendor's perspective, “free” means no charging for the product. From the health system perspective, there is no such thing as free because there’s costs to the institution for implementation, integration, training, and change management.

In health care systems, there’s hundreds of projects going on. There might be a champion in one particular area that’s very excited about something. But then when you think about all the folks that are required to make it a reality, it may involve information technology, clinical, quality, workflow, operations, and legal, among others. Those folks don’t want pilots, they don’t want another thing. There is only so much capacity for change. There’s inertia in the opposite way and so there might be a few people who are excited about it but you got to move an organization there.

There probably needs to be a spectrum regarding deal structure. It depends on the type of product. Smaller products that are closer to a tweak on an existing process will be different than other groundbreaking projects.

Several approaches can be taken to develop real partnerships. Health systems could just get discounted product. They could be paid hourly for consulting. They could earn equity or royalties for your time. They could invest dollars to get equity in the product, with or without a commitment to use the product (Table 4).

**Table 4.** Potential Approaches to Partnerships Between Companies and Health Systems

Pilot (for “free”)
Discounted product
Paid consulting
Product design/inform intellectual property
Fielding referral phone calls
Making connections or introductions
Earn equity
Invest and use product
Invest without commitment to use product
Separate operational from business contributions

How a technology company will approach a health system will depend on where they are in their product development life cycle and their funding situation. Early in its life, a company is looking for validating customers. As a customer, health systems are incredibly valuable to help them prove that their product works, which is needed to secure future sales or funding. So, at different points in their life cycle, they will use different language to imply different things and will have different needs.

There have been venture funds formed on both the health system and the payer side to fund the kinds of products and services that a health system would want and benefit from. If a health system provides funding in addition to revenue, it can also benefit through equity. From the venture capitalist perspective, having a health system investing and buying the service impacts how the business is being funded. Having a health system “eating their own dog chow” is vital for credibility.

It is also important to note that there is often an immediate expectation of insurance coverage without the necessary groundwork. Early pilot work is unlikely to be compensated by third-party payers until there is proven benefit in terms of outcomes.

For health systems with robust research portfolios in the pharmaceutical space, there is a well-established model for new product development. Health systems do not conduct free pilots or clinical trials to determine safety and efficacy of a new medication, while the pharmaceutical company takes it through the Food and Drug Administration (FDA). Pharmaceutical companies raise money to pay health systems to conduct the research. Perhaps we need to move to a model where the value providers and health systems give to these digital health companies is appropriately recognized and built into the early round of raises.

## Prioritizing Decisions

No discussion of ROI can be considered complete without mention of how competing priorities should be handled. Strategic fit guides priorities, but we also acknowledge that emotional and political factors can influence decisions significantly. Achieving a consensus on strategic direction streamlines decision-making and ensures effectiveness in resource allocation.

Balancing new systems, software, and operational needs with ongoing optimizations requires careful planning. Timing is also important. Obviously, within a health system, there is a budget cycle and a capital allocation process. From a benefit design perspective, this also changes on a calendar basis. If you are doing something with the objective of changing the benefit plan, you need to be thinking about when payers shift their benefit design. With respect to self-insured populations, the benefits team is looking at new ideas in the first quarter of the year, but by the fourth quarter, they’re in implementation mode. You can’t talk to them about new ideas. You need to be thoughtful about when you’re planning to do these things.

In conclusion, navigating complex decision-making in health care requires a blend of strategic foresight, practical considerations, and a deep understanding of organizational dynamics. There should not be a specific strategic plan focused on digital or virtual care. The focus should be on the enterprise strategic plan and how can digital enable that. Be opportunistic against the existing strategy and you will be much more likely to deliver value.

## Abbreviations Used

CHF: congestive heart failure
CMS: Center for Medicare and Medicaid
DM: diabetes mellitus
ED: Emergency Department
FDA: Food and Drug Administration
FHIR: Fast Healthcare Interoperability Resources
HTN: hypertension
MRI: magnetic resonance imaging
ROI: return on investment
RPM: remote patient monitoring
